# Auditory Perceptual Abilities Are Associated with Specific Auditory Experience

**DOI:** 10.3389/fpsyg.2017.02080

**Published:** 2017-11-29

**Authors:** Yael Zaltz, Eitan Globerson, Noam Amir

**Affiliations:** ^1^Department of Communication Disorders, Sackler Faculty of Medicine, Tel Aviv University Tel Aviv, Israel; ^2^Jerusalem Academy of Music and Dance Jerusalem, Israel

**Keywords:** auditory training, musicians, auditory experience, psychoacoustic thresholds, frequency discrimination, intensity discrimination, time discrimination, spectrum discrimination

## Abstract

The extent to which auditory experience can shape general auditory perceptual abilities is still under constant debate. Some studies show that specific auditory expertise may have a general effect on auditory perceptual abilities, while others show a more limited influence, exhibited only in a relatively narrow range associated with the area of expertise. The current study addresses this issue by examining experience-dependent enhancement in perceptual abilities in the auditory domain. Three experiments were performed. In the first experiment, 12 pop and rock musicians and 15 non-musicians were tested in frequency discrimination (DLF), intensity discrimination, spectrum discrimination (DLS), and time discrimination (DLT). Results showed significant superiority of the musician group only for the DLF and DLT tasks, illuminating enhanced perceptual skills in the key features of pop music, in which miniscule changes in amplitude and spectrum are not critical to performance. The next two experiments attempted to differentiate between generalization and specificity in the influence of auditory experience, by comparing subgroups of specialists. First, seven guitar players and eight percussionists were tested in the DLF and DLT tasks that were found superior for musicians. Results showed superior abilities on the DLF task for guitar players, though no difference between the groups in DLT, demonstrating some dependency of auditory learning on the specific area of expertise. Subsequently, a third experiment was conducted, testing a possible influence of vowel density in native language on auditory perceptual abilities. Ten native speakers of German (a language characterized by a dense vowel system of 14 vowels), and 10 native speakers of Hebrew (characterized by a sparse vowel system of five vowels), were tested in a formant discrimination task. This is the linguistic equivalent of a DLS task. Results showed that German speakers had superior formant discrimination, demonstrating highly specific effects for auditory linguistic experience as well. Overall, results suggest that auditory superiority is associated with the specific auditory exposure.

## Introduction

A strong linkage between extensive auditory learning and improved auditory perceptual abilities has been demonstrated in a number of prior studies. Specifically, it has been shown that musicians posses superior auditory processing abilities (e.g., Kishon-Rabin et al., 2001; Micheyl et al., 2006; Bidelman et al., 2011; Mandikal Vasuki et al., 2016), and that auditory experience is associated with enhanced neural sound processing (Tervaniemi et al., 2006, 2016; Vuust et al., 2012) as well as structural changes in gray matter (Karpati et al., 2017). These findings are suggested to be a result of long years of intensive training, involving highly demanding processing of different dimensions of the acoustic signal (Zuk et al., 2013; Putkinen et al., 2014). Notwithstanding the strong evidence for an effect of experience on auditory abilities, it is still not clear whether these findings apply to general auditory perceptual abilities, or, rather, to a specific range of aptitudes which are related to a narrow range of professional expertise. A number of prior studies demonstrate that individuals with expertise in the processing of complex auditory information (including professional musicians) show general superiority in the auditory domain (Zuk et al., 2013), including enhanced frequency discrimination (DLF) (Spiegel and Watson, 1984; Koelsch et al., 1999; Kishon-Rabin et al., 2001; Schon et al., 2004; Micheyl et al., 2006; Besson et al., 2007; Schellenberg and Moreno, 2009; Bidelman et al., 2011; Mandikal Vasuki et al., 2016), harmonic sensitivity (Koelsch et al., 2002; Tervaniemi et al., 2005; Musacchia et al., 2008; Zendel and Alain, 2009), timbre sensitivity (Chartrand and Belin, 2006; Sheft et al., 2013; Hutka et al., 2015), and rhythm and meter discrimination (Krumhansl, 2000; Huss et al., 2011; Marie et al., 2011). Intensive auditory experience has also been linked to enhancement in other high level cognitive abilities, such as executive functions (Bialystok and DePape, 2009; Pallesen et al., 2010; George and Coch, 2011; James et al., 2014; Benz et al., 2016; Mandikal Vasuki et al., 2016). An alternative interpretation to these results assumes a different direction of causality, namely that superior auditory performance in musicians originates from a general enhancement in cognitive performance, spanning from executive functions to creativity (for a review, see Benz et al., 2016). Further support for this line of thought comes from behavioral studies demonstrating a superiority of musicians in executive control skills and working memory abilities (e.g., Bialystok and DePape, 2009; Pallesen et al., 2010; George and Coch, 2011; Mandikal Vasuki et al., 2016), as well as objective measures (James et al., 2014). In contrast, other researchers postulate that superior auditory performance is highly specific to the characteristics of the trained auditory skills (Seppänen et al., 2007; Vuust et al., 2012; Tervaniemi et al., 2016).

A number of ERP studies demonstrate that auditory processing advantages exhibited in musicians are limited to a specific range of perceptual abilities, depending on their exact field of expertise (Tervaniemi et al., 2006, 2016; Vuust et al., 2012). Vuust et al. (2012) and Tervaniemi et al. (2016) tested musicians in multiple auditory dimensions, not necessarily associated with their specific field of expertise. Results showed differences in MMN and P3a responses to sound deviants in pitch, timbre, timing, melody, rhythm, or transposition between jazz musicians, rock musicians, and classical musicians. Based on these findings, showing a different auditory “profile” for different musicians, it was suggested that musical training improves auditory performance mainly in the specific auditory characteristics that are relevant to their training (Seppänen et al., 2007; Vuust et al., 2012; Tervaniemi et al., 2016).

Additional support for the specificity of experience-driven superiority in the auditory domain can be found in studies comparing auditory performance in speakers of different native languages, showing superior auditory performance to be related to the specific auditory features of the native language. For example, native speakers of tonal languages, in which pitch contributes to word meaning, were shown to have better interval discrimination (Giuliano et al., 2011), better relative pitch identification ability (Hove et al., 2010), and better pitch discrimination (Pfordresher and Brown, 2009; Giuliano et al., 2011) as compared to English speakers. However, they were not superior to English speakers in auditory abilities that were less relevant to the perception of tonal language, such as timbre discrimination and musical pitch discrimination (Bidelman et al., 2011; Hutka et al., 2015).

Additional evidence supporting the specificity of experience-driven enhancement in the auditory domain can be found in the results of several studies, demonstrating a superiority of Native English speakers in spectrum discrimination (DLS), compared with native speakers of other languages (Kewley-Port et al., 2005; Liu et al., 2012; Mi et al., 2016). English speakers were also shown to have better formant DLF, compared to native Chinese speakers (Liu et al., 2012). This phenomenon was suggested to be the result of a much denser vowel system in English, compared to Chinese. It is important to note that all prior studies which tested the specificity of linguistic-auditory training were conducted using native speakers of *English* as compared to native speakers of other languages.

The possible contradiction between the “auditory-specific” and “general auditory and/or cognitive” superiority models may be attributed, at least in part, to methodological issues. For example, while many studies compared musicians of diverse background to non-musicians, a relative small number of studies focused on sub-groups of musicians, defined by their specific field of expertise. The present study addresses this issue by comparing auditory-experts with different fields of specialty. In the first experiment, a group of pop and rock musicians and a separate group of non-musicians underwent a series of psychoacoustic tasks. These tasks were divided into two main subtypes: those, which tested abilities highly essential for rock and pop musicians, alongside more general psychoacoustic tasks. Results indicating a superiority of musicians’ only in tasks which are directly associated with their area of expertise would provide further support for the model of specificity in auditory learning.

**FIGURE 1:**
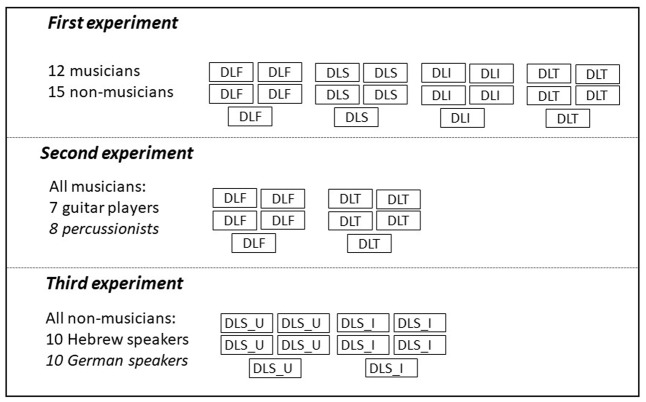
An illustration of the psychoacoustic procedure in all the three experiments. Note that tasks were counterbalanced between participants. DLF, threshold estimate with a difference limen for frequency task; DLS, threshold estimate with a difference limen for spectrum task; DLI, threshold estimate with a difference limen for intensity task; DLT, threshold estimate with a difference limen for time task. DLS_U, threshold estimate with a formant discrimination (linguistic DLS) task with the vowel /u/; and DLS_I, estimate with a formant discrimination task with the vowel /i/.

To follow up on the results of the first experiment, demonstrating a superiority of musicians only in their specific area of expertise, a second experiment was performed, comparing frequency and time discrimination (DLT) between guitar players, who are specifically tuned to pitch differences in their everyday musical experience, and percussionists, who are more tuned to time differences in their everyday experience. Differences between the two groups were expected to be seen only if auditory expertise is exposure-specific. Results partially supported this conjecture. The last experiment examined whether speakers of certain languages would exhibit auditory sensitivities associated with the specific acoustic attributes of their native language. In order to examine this hypothesis, formant discrimination was tested in a group of native German speakers, whose language contains a dense vowel system (Strange and Bohn, 1998), and a group of native Hebrew speakers, whose language has a sparse vowel system (Most et al., 2000). An illustration of the procedure for all three experiments is shown in **Figure 1**. The study was approved by the Institutional Review Board of Tel Aviv University.

## Experiment No. 1

### Participants

Twenty-seven 22- to 35-year-old participants took part in the first experiment: 15 non-musicians (six males), and 12 pop and rock musicians (six males). Musicians were defined as individuals who had at least 8 years of playing experience and at least 1 year of formal musical education. The non-musicians had minimal musical training (less than 1 year of instrumental studies). All participants had pure tone air-conduction thresholds ≤15 dB hearing level bilaterally at octave frequencies from 500 to 4,000 Hz (ANSI, 1996). None of the participants had previous experience in psychoacoustic testing and none had known attention deficits, based on self-report. All participants were naive to the experimental procedure and signed a consent form. Detailed information on the musical background of the musicians is shown in **Table 1**.

**Table 1:** Musical background of the musicians in experiment no. 1.

Participant number	Academic education	Musical instrument	Onset of playing	Years of playing
1	First year student in the musical academy of Tel-Aviv university	Piano and small organ	7	15
2	First year student in ‘Rimon’ private school of music	Keyboard and tempo instruments	15	10
3	First year student in the musical academy of Tel-Aviv university	Cello	5	17
4	Fourth year graduated in ‘Muzik’ private school of music	Piano and guitar	8	20
5	MA from the musical academy of Tel-Aviv university	Accordion and piano	12	20
6	Third year graduate from ‘Rimon’ private school of music	Guitar	13	20
7	Third year graduate from ‘Levinski’ college in the course of B.Ed. in music education	Piano	5	19
8	First year student in ‘Rimon’ private school of music	Guitar	16	9
9	First year student in ‘Rimon’ private school of music	Piano, flute and singing	16	12
10	Third year graduate from ‘Rimon’ private school of music. Fourth year student in ‘Levinski’ college in the course of B.Ed. in music education	Guitar, harmonica and singing	18	12
11	Second year graduate from ‘Rimon’ private school of music	Flute and singing	8	15
12	Fourth year graduate in the musical academy of Jerusalem university	Piano and singing	6	21


### Stimuli

Stimuli were digitally generated at a sampling rate of 22,050 Hz and 16-bits using Matlab software. For the DLF task: stimuli consisted of 1,000–1,200 Hz pure-tones that varied in 1 Hz steps. For the intensity discrimination (DLI) task: stimuli consisted of 1,000 Hz pure-tones spanning an intensity range of 20 dB, varying in 0.1 dB steps. For the DLS task: stimuli consisted of complex tones with 11 harmonics spaced 200 Hz from each other, spanning 200–2,000 Hz. The spectral envelope of the stimuli was a straight line varying in slope from 0 to -20 dB/octave, in steps of 0.1 dB/octave. Stimuli for these three tasks had a total duration of 300 ms and were gated with rise and fall time cosine ramps of 25 ms. For the DLT task: stimuli included pairs of drumbeats separated by silence intervals corresponding to a range of tempos of 160–80 beats per minute (BPM) from 0.375 to 0.75 s. Steps were 0.4 BPM. Stimuli were delivered from a personal computer through an A177 PLUS audiometer and via PELTOR H74 earphones.

### Procedure

Each participant took part in a single testing session that lasted approximately 2 h. Testing included overall 20 thresholds measurements, 5 measurements in each of the four tasks: DLF, DLI, DLT, and DLS. These were counterbalanced. A few minutes break was given between tasks, on demand. Testing was conducted in a quiet room.

### Thresholds Measurement

Thresholds were evaluated using a three-interval, two-alternative, forced choice (3I2AFC) adaptive procedure. Each trial consisted of three stimuli: two reference tones and one comparison tone. The first stimulus in each trial was always the reference tone and the comparison tone was presented randomly as either the second or the third in the sequence. The comparison tone was always the higher (for the DLF and DLS task)/stronger (for the DLI task)/longer (for the DLT task) than the other two. The stimuli were presented simultaneously with three rectangular numbered buttons on the computer screen. Button No. 1 was grayed out, since it could not be pressed. Participants were instructed to identify the tone that was different and use the mouse to click the appropriate button. There was no time limit for the response and no feedback was provided. A two-down, one-up tracking procedure was used in order to estimate the 70.7% correct point on the psychometric function (Levitt, 1971). Each threshold measurement ended after 10 reversals (turn-points) at the minimum step size or after 200 stimuli. Thresholds were calculated as the geometric mean of eight turn-points at minimal step size. Before the first threshold’s assessment in each task, a short familiarization with the task was conducted with the easiest discriminated stimuli [with a difference of 200 Hz for the DLF task, 20 dB for the DLI task, 80 BPM for the DLT task, and (-20) dB/Octave for the DLS task], until five successively correct answers were provided. No feedback was provided during testing. For the DLF task, initial step size was 200 Hz and it was reduced by half every turn-point until reaching a minimal step size of 1 Hz. For the DLI task, initial step size was 20 dB and it was reduced by half every turn-point until reaching a minimal step size of 0.1 dB. For the DLT task, initial step size was 80 BPM and it was reduced by half every turn-point until reaching a minimal step size of 0.48 BPM. For the DLS task, initial step size was 20 dB/Octave and it was reduced by half every turn-point until reaching a minimal step size of 0.1 dB/Octave.

### Data Analysis

The data were log-transformed in order to avoid a violation of the homoscedasticity assumption of the parametric statistical tests and to normalize the distribution of the variables (Kolmogorov–Smirnov test: *p* > 0.05). The employment of a logarithmic transformation was also motivated by the nature of auditory perception, which is logarithmic in nature (Moore, 2003).

Four two-way analyses of variance (ANOVA) with repeated measures were conducted, separately for each task with group as the between-subjects factor and measurement (1–5) as the within-subject factor, with adjustments for multiple comparisons. *Post hoc* pairwise comparisons with Bonferroni corrections were used for significant interactions. Pearson correlation tests were conducted among the mean thresholds of the different tasks, separately for each group, and between years of musical experience and thresholds in the different tasks for the musicians group.

### Results

**FIGURE 2:**
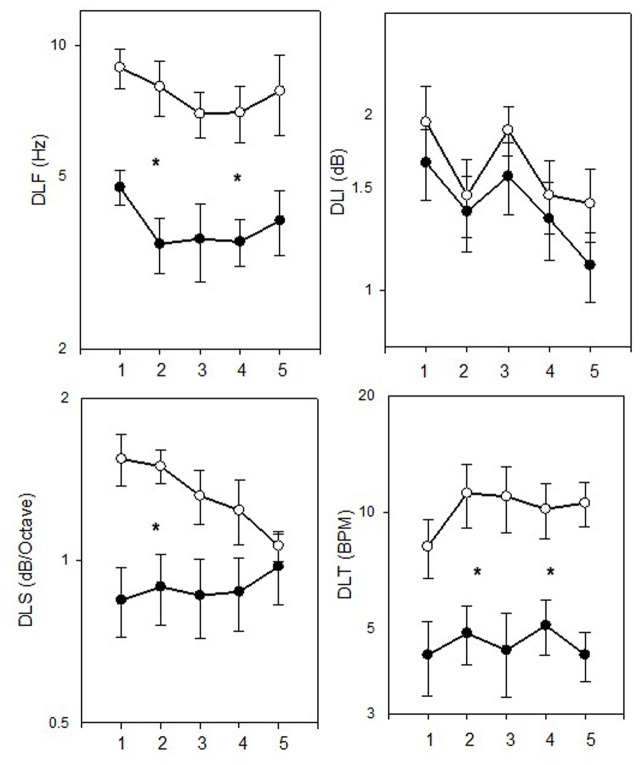
Mean thresholds (±SE) in the DLF, DLI, DLS, and DLT tasks for the musicians (filled symbols) and non-musicians (empty symbols). Asterisks represent significant (*p* < 0.05) difference.

Thresholds in the four tested tasks (DLF, DLI, DLS, and DLT) are shown in **Figure 2**, separately for the musicians and non-musicians. Data suggest a different pattern of behavior in each task, with musicians showing consistently superior performance only in the DLF and DLT tasks. Specifically, in the DLF task, better thresholds were shown for the musicians (*M* = 3.85 ± 1.96 Hz) as compared to the non-musicians (*M* = 7.74 ± 4.42 Hz) [*F*(1,25) = 13.656, *p* = 0.001, η^2^ = 0.353], with a significant difference between the measurements [*F*(4,25) = 4.006, *p* = 0.006, η^2^ = 0.138]. Significant linear and quadratic effects were evident between the measurements [*F*(1,25) = 8.071, *p* = 0.009; *F*(1,25) = 8.042, *p* = 0.009, respectively], with no significant group × measurement interaction, indicating that both groups improved with testing. In the DLI task, similar performance was shown for the musicians (*M* = 1.41 ± 0.71 dB) and non-musicians (*M* = 1.63 ± 0.81 dB) [*F*(1,25) = 0.817, *p* = 0.375], and significant difference was shown between the measurements [*F*(4,25) = 4.529, *p* = 0.002, η^2^ = 0.153]. Significant linear and cubic effects were shown between the measurements [*F*(1,25) = 7.862, *p* = 0.010; *F*(1,25) = 5.307, *p* = 0.030, respectively], with no significant group × measurement interaction, indicating that both groups improved with testing. In the DLS task, better thresholds were shown for the musicians (*M* = 0.89 ± 0.41 dB/octave) as compared to the non-musicians (*M* = 1.33 ± 0.55 dB/octave) [*F*(1,25) = 10.355, *p* = 0.004, η^2^ = 0.293], with no significant difference between the measurements [*F*(1,25) = 1.173, *p* = 0.327]. A border-line significant group × measurement interaction [*F*(4,25) = 2.369, *p* = 0.058, η^2^ = 0.087] revealed, however, that the musicians reached better thresholds than the non-musicians only in the first three measurements (*p* < 0.022). A border-line significant linear effect between the measurements [*F*(1,25) = 3.628, *p* = 0.068], with significant group × measurement interaction for this linear effect [*F*(1,25) = 10.123, *p* = 0.004] further indicated that only the non-musicians improved with testing. In the DLT task, better thresholds were shown for the musicians (*M* = 4.58 ± 2.94 BPM) as compared to the non-musicians (*M* = 10.21 ± 6.74 BPM) [*F*(1,25) = 16.071, *p* < 0.001, η^2^ = 0.391], with no significant difference between the measurements [*F*(1,25) = 1.892, *p* = 0.118], and no significant group × measurement interaction [*F*(1,25) = 0.724, *p* = 0.578].

**FIGURE 3:**
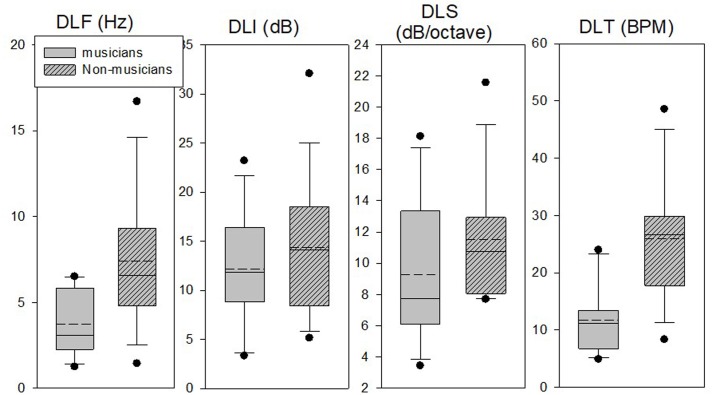
Box plots of the mean last two measurements (4 and 5) in the DLF, DLI, DLS, and DLT tasks for the musicians and non-musicians. Box limits include the 25th–75th percentile data. Continuous line within the box represents the median. Dashed line within the bars represents mean. Bars extend to the 10th and 90th percentiles. Black dots represent outliers.

Due to the significant effect of measurement found for some of the tasks, we examined the scatter of only the last two measurements (mean of measurements 4 and 5) in each task, using box and whiskers plots (**Figure 3**). Results strengthened the previous analysis by showing that in the DLF and DLT tasks the majority of the musicians did better than the non-musicians, and in the DLI and DLS there was a large overlap between the groups. It was also shown, however, that in both the DLF and DLT tasks there were some non-musicians (about 10–15% of the group) who reached thresholds that were similar to the musician’s mean thresholds.

**FIGURE 4:**
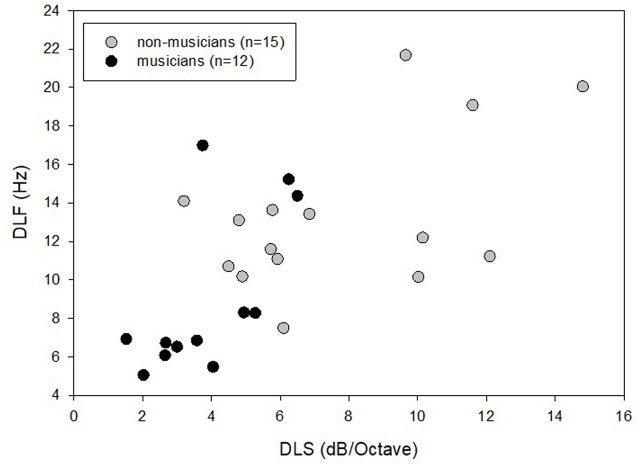
Individual DLF and DLS thresholds of the non-musicians and musicians.

Pearson correlation tests revealed a significant association between the mean thresholds in the DLF task and the mean thresholds in the DLS task only for the non-musicians (*r* = 0.51, *p* = 0.031). A scatter plot of the mean DLF vs. DLS thresholds for the non-musicians and musicians is shown in **Figure 4**. It can be seen that larger between-subject variance was evident for the non-musicians (DLF thresholds ranged from 3.2 to 14.8, DLS thresholds ranged from 7.5 to 21.68) as compared to the musicians (DLF thresholds ranged from 1.53 to 6.5, DLS thresholds ranged from 5.07 to 17). No other associations between the tasks were found significant (*p* > 0.05). No significant associations were found between musician’s experience or age of onset of musical training (in years) and mean thresholds in any of the tested tasks (*p* > 0.05), possibly because all the musicians had extensive musical experience (more than 8 years).

### Discussion

The results of the first experiment show that pop and rock musicians are superior to non-musicians only in some psychoacoustic tasks. Specifically, while the musicians outperformed the non-musicians in the DLF and DLT tasks throughout the five threshold measurements, musician’s superiority was less marked for the DLS task (with both groups reaching similar performance by the last two measurements) and no musician’s superiority was shown for the DLI task.

The finding that musicians have better frequency and DLT abilities as compared to non-musicians is supported by previous behavioral studies, which tested each dimension separately (Spiegel and Watson, 1984; Kishon-Rabin et al., 2001; Tervaniemi et al., 2005; Micheyl et al., 2006; Bidelman et al., 2011; Banai et al., 2012; Mishra et al., 2014, 2015). These findings may be in favor of the hypothesis that musicians have better processing of subtle acoustic information at the sensory level (Banai et al., 2012), highlighting enhanced sensitivity to temporal fine structure (Mishra et al., 2015) alongside enhanced temporal resolution ability (Mishra et al., 2014). Previous studies also suggested that trained musicians may have higher perceptual acuity for timbre characteristics as compared to non-musicians (Chartrand and Belin, 2006; Sheft et al., 2013; Hutka et al., 2015). Our current findings, on the other hand, show that while there was some advantage for the musicians as compared to the non-musicians in the first few measurements of the DLS task, which represents sensitivity to timbre, it quickly faded away. This controversy may possibly be explained by the different protocol of testing between the studies. While previous studies testing timbre perception were generally based on short exposure to the tested task (which included one to two measurements), the present study included longer exposure of five measurements for the task. Therefore, we were able to show that the non-musicians needed only a short practice in order to “close the gap” and reach as good timbre sensitivity as the musicians. The musician’s superiority in the first few measures of the DLS may have reflected, therefore, a general sensitivity to changes in spectrum for musicians, or a general enhancement in executive functions, rather than a genuine, sensory advantage on the task. Interestingly, a significant correlation between DLS and DLF was found only in the non-musician group. This can be explained to some extent by the larger variability shown by this group in their DLF and DLS thresholds. At this point, it is difficult to determine why a significant correlation was found only between these two tasks.

Finally, the lack of difference between musicians and non-musicians in the DLI task may suggest that in contrast to the other tested auditory sensitivities, rock music experience does not improve sensitivity to intensity changes. This finding may further support the notion that superior auditory performance does not stem from a general enhancement in top-down cognitive mechanisms, such as improved auditory attention and enhanced short-term memory traces (Strait et al., 2010). Had this been the case, one would expect the musicians to be better than the non-musicians in all the tested auditory tasks. Rather, a consistent musician’s superiority was exhibited only in the sensitivity to time and frequency changes. Rhythm and pitch are the two primary dimensions of music of any culture (Krumhansl, 2000). Using time and pitch changes, complex musical patterns are constructed, accompanied by highly complex psychological representations (Krumhansl, 2000). Hence, any musician, across culture and musical style, must develop an acute sensitivity to these basic acoustic components of sound. Furthermore, pop music is generally characterized by high degrees of loudness, explaining the lack of differences in DLI between the musicians and non-musicians. Somewhat different results, however, might have been found for classical musicians.

## Experiment No. 2

In order to further refine the results of the first experiment, we tested the effect of specific expertise on auditory perceptual abilities in musicians. The DLF and DLT tasks were chosen, since they were found to be different between musicians and non-musicians in the first experiment. Two groups of musicians were chosen to participate in this experiment: guitar players, who tune their instruments, and thus must specialize in pitch discrimination, and percussionists, who do not need to tune their (unpitched) instruments, but, on the other hand, are required to follow highly complex rhythmic patterns.

### Procedure

A second group of 15 18- to 32-year-old pop and rock musicians, eight guitar players, and seven percussionists took part in this experiment (male = 14). Musicians were defined as individuals who had at least 8 years of playing experience and at least 1 year of formal musical education. Detailed information about their musical background is shown in **Table 2**. None of these musicians had participated in the first experiment. All participants were naive to the experimental procedure and signed a consent form. Other criteria for inclusion in the study were similar to the first experiment. Stimuli and testing conditions were identical to the first experiment. A 3I2AFC adaptive procedure was used to estimate thresholds in the DLF and DLT tasks, similarly to the first experiment, using two-down one-up adaptive tracking procedure. Participants were tested in a single session that included 10 measurements, five in each task, and lasted approximately 1 h. The order of tasks was counterbalanced between participants.

**Table 2:** Musical background of the musicians in experiment no. 2.

Participant number	Academic education	Musical instrument	Years of playing
1	Graduate of the faculty of music in Bar-Ilan university	Guitar	18
2	Third year student in ‘Rimon’ private school of music	Guitar	13
3	Graduate of the music school of Ono academic college	Guitar	16
4	Second year student of ‘Rimon’ private school of music	Guitar	8
5	Graduate of ‘Muzic’ private school of music	Guitar	15
6	Third year student in ‘Rimon’ private school of music	Guitar	13
7	Servicing in the army as a player in the military band	Guitar	16
8	Graduate of the faculty of music in Bar-Ilan university	Guitar	19
9	Second year student in ‘Drummer’ private school of music	Percussion	9
10	Graduate of ‘Rimon’ private school of music	Percussion	12
11	Graduate of ‘Drummer’ Private school of music	Percussion	24
12	Graduate of ‘Rimon’ private school of music	Percussion	14
13	Third year student in ‘Levinski’ academic college in the course of B.Ed. in music education	Percussion	12
14	Graduate of ‘Muzic’ private school of music	Percussion	20
15	Graduate of ‘Rimon’ private school of music	Percussion	17


### Data Analysis

Two two-way ANOVAs with repeated measures were conducted separately on the DLF and DLT results of the musicians from the second experiment and the non-musicians from the first experiment with group (musicians, non-musicians) as the between-subject variable and measurement (1–5) as the within-subject variable. Two more two-way ANOVAs with repeated measures were separately conducted on the DLF and DLT results of the musicians from the second experiment with group (guitar players, percussionists) as the between-subject variable and measurement (1–5) as the within-subject variable.

### Results

In order to test whether the group of musicians that was tested in this experiment had superior DLF and DLT performance, as predicted by the results of the first experiment, we compared the musician’s results, as a group (*n* = 15), to that of the non-musicians in Experiment 1, using two two-way ANOVAs. Results confirmed better DLF and DLT thresholds for the musicians as compared to the non-musicians [*F*(1,28) = 14.812, *p* = 0.001 for the DLF; *F*(1,28) = 9.029, *p* = 0.006 for the DLT].

**FIGURE 5:**
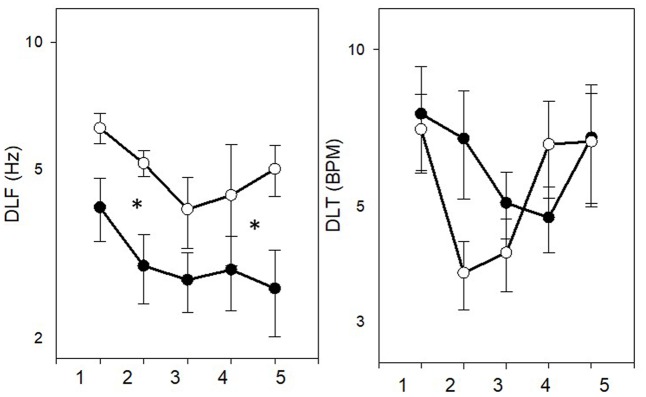
Mean thresholds (±SE) in the DLF and DLT task for guitar players (filled symbols, *n* = 8) and percussionists (empty symbols, *n* = 7). Asterisks represent significant (*p* < 0.05) difference.

**Figure 5** shows the DLF and DLT thresholds of the guitar players versus those of the percussionists. ANOVA analyses showed that in the DLF task, the guitar players (*M* = 3.07 ± 1.63 Hz) were significantly better than the percussionists (*M* = 4.97 ± 2.19 Hz) [*F*(1,13) = 6.284, *p* = 0.026, η^2^ = 0.326], and there was a significant difference between the measurements [*F*(1,13) = 3.935, *p* = 0.019]. A significant linear effect was shown between the measurements [*F*(1,13) = 10.715, *p* = 0.006] with no significant effect × music type interaction, indicating significant improvement with testing for both groups. In the DLT task, on the other hand, the guitar players (*M* = 5.86 ± 3.9 BPM) were slightly inferior to the percussionists (*M* = 5.09 ± 2.42 BPM), though this difference was not found significant [*F*(1,13) = 0.215, *p* = 0.615]. Similarly to the previous experiment, no significant difference was shown between the measurements [*F*(1,13) = 2.745, *p* = 0.122], and no significant music type × measurement interaction was found [*F*(1,13) = 2.103, *p* = 0.094].

**FIGURE 6:**
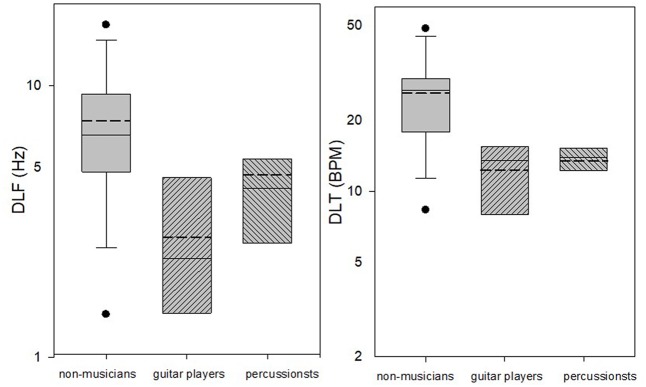
Box plots of the mean last two measurements (4 and 5) in the DLF and DLT tasks of the guitar players and percussionists (second experiment) compared to the non-musicians (first experiment). Box limits include the 25th–75th percentile data. Continuous line within the box represents the median. Dashed line within the bars represents mean. Bars extend to the 10th and 90th percentiles. Black dots represent outliers.

**Figure 6** shows box and whisker plots for the thresholds of the guitar players and percussionists as compared to the thresholds of the non-musicians in the first experiment. Since there was a significant improvement with testing for the DLF task, only the mean of the last two measurements (4 and 5) is shown. Results show that in the DLF task, as expected from the ANOVA results, the guitar players reached better mean and median results as compared to the percussionists, though there was some overlap between the groups, suggesting that several percussionists reached as good thresholds as the guitar players. In the DLT task, on the other hand, the mean and median of the percussionists’ results were similar to that of the guitar player’s, with a larger range of results for the guitar players. In addition, both groups did better than the non-musicians in the DLF and DLT tasks though some non-musicians reached “musician’s thresholds” in both tasks. These results demonstrate a between-subjects variance in all three groups, irrespective of musical experience.

### Discussion

The results of the second experiment demonstrate superior DLF abilities in guitar players, compared to percussionists. These findings add to the outcomes of the previous experiment by suggesting that not only does the superior auditory performance of musicians depend on the tested auditory task, but it also depends on their specific musical field of expertise. Tuning a guitar requires high sensitivity to miniscule differences in pitch. DLF ability is, therefore, exercised repeatedly over years of playing and is essential for an expert guitar player. On the other hand, rock and pop percussion players mostly perform on unpitched instruments, and are therefore not required to develop a high sensitivity to small pitch differences. The present findings are therefore in line with recent ERP studies showing a different auditory “profile” for different musicians, depending on their music style/genre (Vuust et al., 2012; Tervaniemi et al., 2016).

The finding that the percussionists were not different from the guitar players in their DLT thresholds is not entirely surprising. Given that percussionists specialize in performing highly complex rhythmic patterns, one could expect them to demonstrate superior temporal discrimination abilities. However, guitar players as well as other pitched instrument players are also required to demonstrate high proficiency in rhythm perception and production. Another possible interpretation of these results is that since the DLT task evaluated the lowest level of rhythm perception (i.e., sensitivity to onset timing), this task did not directly apply to higher hierarchical levels of rhythmic patterns employed in music performance. Hence, future studies are required to further evaluate temporal perception in these two groups of instrumentalists.

Interestingly, both groups of musicians, i.e., guitar players and percussionists, demonstrated superior performance on both frequency and DLT tasks, in comparison to non-musicians. Moreover, the fact that both musician groups performed better on the DLT task than non-musicians is understandable. Both groups of musicians must perform within the rhythmic framework of a musical piece, and coordinate their rhythmic performance. Thus, although the DLT task in the present study may not have been sensitive enough to reflect high-level rhythmic processing differences between guitar players and percussionists, it was sensitive enough to reflect the better time processing of both groups as compared to non-musicians. On the other hand, it is less trivial that percussionists are better than non-musicians on the DLF task. A possible explanation is that long hours of listening and participating in musical performances result in improved auditory capabilities in the domain of pitch perception, yet not as developed as those observed in non-percussive instruments. This explanation is further supported by the results of a recent study showing that non-musicians who listen regularly to classical Arab-music, which employs small pitch intervals of quartertones, have better pitch discrimination abilities, compared to Western-music listeners (Globerson et al., 2016).

## Experiment No. 3

In the final experiment, we tested whether a specific linguistic background may also result in specific auditory superiority. Native German speakers, whose language contains a dense vowel system were compared to native Hebrew speakers, whose language has a sparse vowel system, using a linguistic DLS task, i.e., a formant discrimination task.

### Participants

Twenty 18- to 27-year-old non-musicians (nine males) took part in this experiment forming two groups: 10 (three males) native Hebrew speakers and 10 (six males) native German speakers. None of the participants was exposed to the other language on a daily basis. All participants were naive to the experimental procedure and signed a consent form. Other criteria for inclusion in the study were similar to the first experiment.

### Stimuli

**FIGURE 7:**
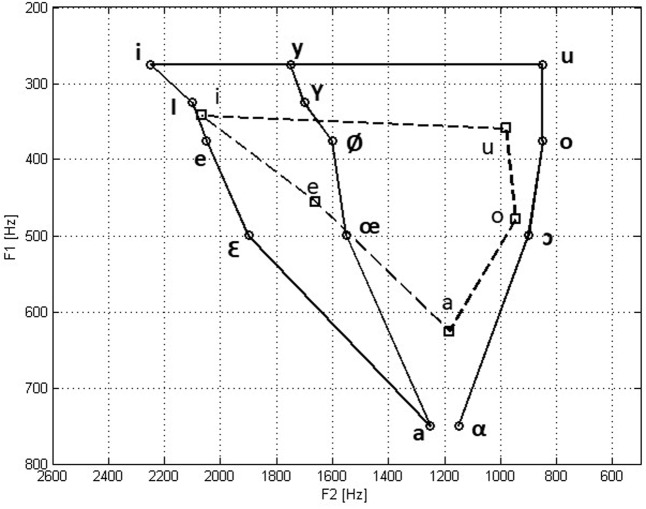
An illustration of the vowel space between the first and second formants for German (continues line) and Hebrew (dashed line).

The main rationale of the experiment was based on the difference in vowel density between the languages. While Hebrew consists of 5 vowels, the German language includes 14 vowels. An illustration of the vowel space is shown in **Figure 7** for the two languages (Delattre, 1964; Most et al., 2000).

Stimuli were synthesized at a sampling rate of 8,000 Hz and 16-bit using custom-written Matlab software. Synthesis was based on analysis of /i/ and /u/ as uttered by a native German speaker and measured with Praat software, giving the following values: For the reference /i/ vowel: f1 = 216 Hz, f2 = 2306 Hz; for the reference /u/ vowel: f1 = 295 Hz, f2 = 740 Hz. All stimuli were synthesized with a fundamental frequency of 120 Hz, which was the approximate mean f0 of the original speaker. Synthesis of each stimulus was performed by passing an impulse train through an all pole filter approximating the vocal tract with the desired f1 and f2 values. The synthesized /i/ vowels varied in their second formant from 2,306 Hz down to 2,006 Hz in 200 steps of 1.5 Hz, and the synthesized /u/ vowels varied in their second formant from 740 Hz up to 980 Hz in 200 steps of 1.2 Hz. Thus, both series of stimuli started with the original f2 value and shifted gradually to more central f2 values. Each stimuli lasted 300 ms.

### Thresholds Measurement

Formant discrimination thresholds were evaluated using the same procedure used in the first two experiments (3I2AFC). Initial step size was for the /i/ vowel 300 Hz and 260 Hz for the /u/ vowel.

### Procedure

A 3I2AFC adaptive procedure was used to estimate thresholds in the formant discrimination (linguistic DLS) task, similarly to the first two experiments, using two-down one-up adaptive tracking procedure. Participants were tested in a single session that included 10 measurements, five in each vowel and lasted approximately 1 h. The order of vowel presentation was counterbalanced. Testing conditions were similar to the first two experiments.

### Data Analysis

Three-way ANOVA with repeated measures was conducted on the formant discrimination thresholds with group (native German speakers, native Hebrew speakers) as the between-subject variable and vowel (/i/, /u/) and measurement (1–5) as the within-subject variables.

### Results

**FIGURE 8:**
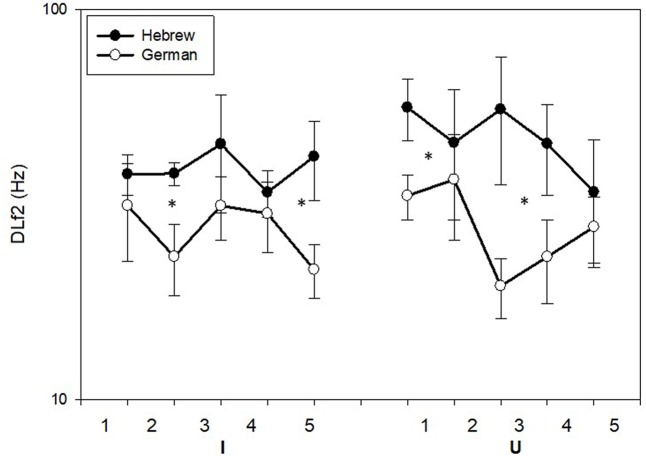
Mean thresholds (±SE) of f2 in the formant discrimination task for the native Hebrew speakers (*n* = 10) and native German speakers (*n* = 10). Asterisks represent significant (*p* < 0.05) difference.

The thresholds of the German speakers and Hebrew speakers in the formant discrimination task are shown in **Figure 8** separately for the /i/ and /u/ vowels. Statistical analysis revealed significant difference between the German speakers and the Hebrew speakers [*F*(1,20) = 6.235, *p* = 0.021, η^2^ = 0.238] with the German speakers reaching better discrimination thresholds. Significant difference was shown between the measurements [*F*(4,20) = 4.482, *p* = 0.003, η^2^ = 0.183], with significant linear effect (*p* < 0.001) showing both groups to improve between the measurements. No significant difference was shown between the two vowels [*F*(1,20) = 0.025, *p* = 0.876] nor were there any significant interactions (*p* > 0.05).

**FIGURE 9:**
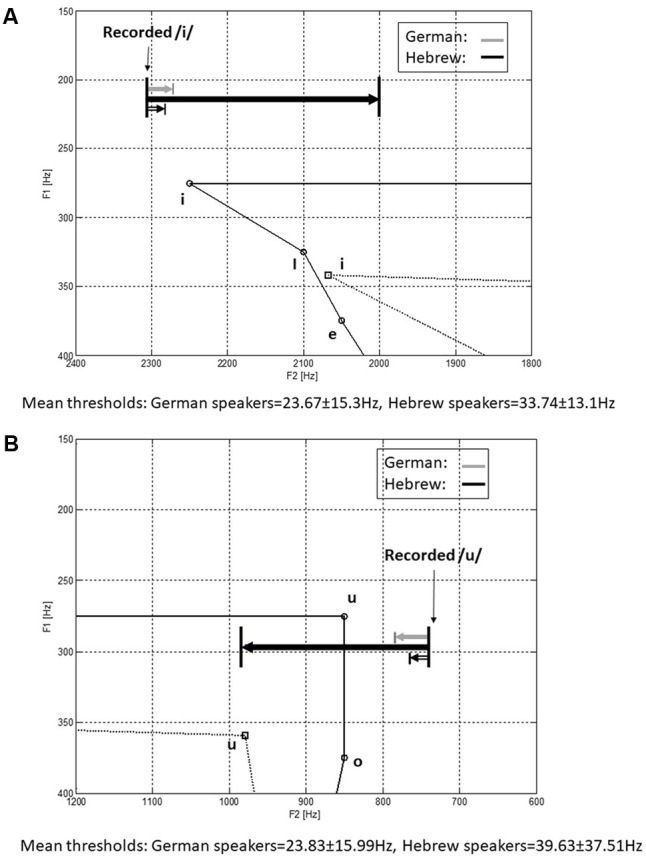
Mean formant discrimination thresholds in the last two measurements (4 and 5), with vowels /i/ **(A)** and /u/ **(B)** of the native Hebrew speakers and native German speakers, with respect to the f1 and f2 values of the recorded vowels and the range of tested differences. Also shown is the illustration of the relevant vowel density in German and Hebrew.

Since there were significant improvements between measurements, **Figure 9** shows the mean formant discrimination thresholds of the last two measurements with /i/ (**Figure 9A**) and /u/ (**Figure 9B**) for the Hebrew and German speakers. Also shown are the f1 and f2 values of the recorded vowels and the range of f2 variation over the adaptive threshold measurement. It can be seen that the formant values of the German speaker recorded here deviated slightly from the reported mean in the German language (Delattre, 1964). Nevertheless, even after a short practice (measurements 1–3) the German speakers demonstrated greater sensitivity to changes in f2, than the Hebrew speakers.

### Discussion

The results of the third experiment suggest that auditory sensitivity to formant differences is improved in individuals who are experienced in perceiving and producing fine formant differences. That is, individuals who hear and speak a language characterized by finer differences between vowels, such as German, develop better formant DLF over time, compared to individuals who are exposed to a language that has larger differences between its vowels, such as Hebrew. These results add to the results of the previous experiments by showing that specificity in auditory superiority can also take place following specific linguistic experience. Previous studies that tested formant DLF by comparing native English speakers to native speakers of other languages further support these findings (Kewley-Port et al., 2005; Liu et al., 2012; Mi et al., 2016). Future studies could test whether superior formant discrimination performance stemming from linguistic exposure carries over to non-linguistic stimuli as well. For example, it might be worthwhile to examine DLS for non-linguistic stimuli in addition to formant discrimination in native speakers of languages that have different vowel systems, given that both tasks represent sensitivity to timbre, though one is musical and the other is linguistic.

The results of the third experiment also emphasize the fact that auditory experience does not have to include formal training in order to affect auditory perception. Linguistic experience is a life-long ongoing process, beginning with the first day of life, while formal music training typically begins later in childhood. Moreover, “linguistic experience” does not include focused attention on the acoustic characteristics of the speech stimuli but rather on communicational goals. Despite these differences, linguistic experience was found to be linked with perception of relevant acoustic features in individuals with no musical expertise.

## General Discussion

Overall, the results of the experiments in this study emphasize the effect of active and passive experience on auditory perceptual abilities. In the first experiment, experienced pop and rock musicians showed superior auditory abilities over non-musicians only in tasks directly related to their genre of music. In the second experiment, musicians of different backgrounds were found to differ in their auditory sensitivity to frequency differences, in accordance with their field of expertise, though they were not found to differ in their sensitivity to time differences. It is possible that musical background affected only frequency and not time sensitivity since both musician groups were well trained in rhythm perception and production. Alternatively, it is possible that the specific task used to assess time perception in the present study did not reflect high rhythm perception levels, and thus may have failed to detect any fine differences in complex temporal perception between the musician groups. In the third experiment, superior formant discrimination ability was shown for non-musician, native German speakers, who are exposed on a daily basis to a vowel system, which relies on fine formant discrimination ability. The use of the same protocol of testing in all three experiments, varying in the specific tested tasks and populations, allowed a controlled examination of the effects of experience on auditory perceptual abilities. Taken together, results suggest a possible dependency of auditory perceptual learning on the exact characteristics of prior experience. Hence, auditory learning appears to have a selective effect on perception, which may also be reflected in a selective enhancement in the underlying sensory mechanisms. The results of the current study may provide meaningful insights regarding auditory training in clinical populations. For example, musical training with an instrument, which needs tuning, may improve sensitivity to pitch in children who experience poor spectral processing, such as hearing-impaired children with cochlear implants (Henry et al., 2005; Winn et al., 2016). Training with percussion instruments may benefit dyslectics, who have been shown to have deficiencies in some basic temporal abilities (Overy, 2003; Huss et al., 2011; Flaugnacco et al., 2014). Overall, a high level of specificity in auditory training could substantially improve the effect of such interventions in clinical populations.

Future studies may wish to test a broader range of auditory abilities in order to strengthen our conclusion, regarding the specific effects of auditory experience on auditory perception. The present study tested differences in auditory perception between specific groups that varied in musical background, and specific groups that varied in linguistic background. Testing more groups, including a broader range of linguistic and musical backgrounds, may be advised in order to further support our results and generalize them to different populations.

## Author Contributions

YZ, EG, and NA had substantial contributions to the conception or design of the work, i.e., to the acquisition, analysis, and interpretation of data for the work. They also contributed to drafting the work and revising it critically for important intellectual content. They all gave their final approval of the version to be published and agreed to be accountable for all aspects of the work in ensuring that questions related to the accuracy or integrity of any part of the work are appropriately investigated and resolved.

## Conflict of Interest Statement

The authors declare that the research was conducted in the absence of any commercial or financial relationships that could be construed as a potential conflict of interest.
